# Asymptomatic neurotoxicity of amyloid β-peptides (Aβ_1-42_ and Aβ_25-35_) on mouse embryonic stem cell-derived neural cells

**DOI:** 10.17305/bjbms.2020.4639

**Published:** 2021-02

**Authors:** Nur Izzati Mansor, Carolindah Makena Ntimi, Noraishah Mydin Abdul-Aziz, King-Hwa Ling, Aishah Adam, Rozita Rosli, Zurina Hassan, Norshariza Nordin

**Affiliations:** 1 Genetics and Regenerative Medicine Research Centre, Faculty of Medicine and Health Sciences, Universiti Putra Malaysia, Serdang, Selangor, Malaysia; 2 Medical Genetics Unit, Department of Biomedical Science, Faculty of Medicine and Health Sciences, Universiti Putra Malaysia, Serdang, Selangor, Malaysia; 3 Department of Parasitology, Faculty of Medicine, University of Malaya, Kuala Lumpur, Malaysia; 4 Pharmacology and Toxicology Research Laboratory, Faculty of Pharmacy, Puncak Alam Campus, Universiti Teknologi MARA, Shah Alam, Selangor, Malaysia; 5 UPM-MAKNA Cancer Research Laboratory, Institute of Bioscience, Universiti Putra Malaysia, Serdang, Selangor, Malaysia; 6 Centre for Drug Research, Universiti Sains Malaysia, Gelugor, Penang, Malaysia

**Keywords:** Amyloid β-peptides, Alzheimer’s disease, reactive oxygen species, oxidative stress, 46C mouse embryonic stem cell

## Abstract

One of the strategies in the establishment of *in vitro* oxidative stress models for neurodegenerative diseases, such as Alzheimer’s disease (AD), is to induce neurotoxicity by amyloid beta (Aβ) peptides in suitable neural cells. Presently, data on the neurotoxicity of Aβ in neural cells differentiated from stem cells are limited. In this study, we attempted to induce oxidative stress in transgenic 46C mouse embryonic stem cell-derived neurons via treatment with Aβ peptides (Aβ_1-42_ and Aβ_25-35_). 46C neural cells were generated by promoting the formation of multicellular aggregates, embryoid bodies in the absence of leukemia inhibitory factor, followed by the addition of all-trans retinoic acid as the neural inducer. Mature neuronal cells were exposed to different concentrations of Aβ_1-42_ and Aβ_25-35_ for 24 h. Morphological changes, cell viability, and intracellular reactive oxygen species (ROS) production were assessed. We found that 100 μM Aβ_1-42_ and 50 μM Aβ_25-35_ only promoted 40% and 10%, respectively, of cell injury and death in the 46C-derived neuronal cells. Interestingly, treatment with each of the Aβ peptides resulted in a significant increase of intracellular ROS activity, as compared to untreated neurons. These findings indicate the potential of using neurons derived from stem cells and Aβ peptides in generating oxidative stress for the establishment of an in vitro AD model that could be useful for drug screening and natural product studies.

## INTRODUCTION

The two main lesions found in the brain of Alzheimer’s disease (AD) patients are 1) senile plaque that is composed of amyloid beta (Aβ) peptides and 2) neurofibrillary tangles that are composed of the hyperphosphorylated tau protein. Senile plaques first develop in the brain neocortex followed by the hippocampus, and then they spread to all regions of the brain in the centripetal motion [1]. This lesion is due to the deposition of Aβ peptides in the cells. Aβ peptides (~4 kDa) are an abnormal proteolytic by-product of the transmembrane protein amyloid precursor protein (APP) [2,3]. The *APP* gene is expressed on chromosome 21 in the synapses of neurons, and it is important for neuronal development [4,5]. In the healthy brain, APP undergoes alternative splicing in which APP is cleaved by α-secretase at or adjacent to lysine-16 in the Ab sequence, on the surface of neurons. Successively, the C-terminal site of soluble APP-alpha is released from the membrane and secreted from the cell, hence reducing the deposition of Aβ protein in the cells. On the other hand, in the case of AD, APP is cleaved by β- and γ-secretase, which subsequently produces several isoforms of amino acid residues ranging from 39 to 43 residues [6-10] and called Aβ peptides.

The accumulation of Aβ peptides in the brain potentially can 1) generate reactive oxygen species (ROS); 2) initiate the apoptosis cascades; and 3) promote neurotoxicity [11-15]. Previous studies have shown that Aβ peptides cause dendritic degeneration and synapse loss in the rat hippocampus [16,17], signifying the neuronal death. Besides, the neurotoxicity of Aβ peptides in the form of extracellular fibrillar aggregates *in vitro* has been well-documented in the previous studies using either primary neuronal cells or cancerous cell lines, such as those derived from rat pheochromocytoma, PC12 cells, and human neuroblastoma, SH-SY5Y cells [14,18-21]. The major limitations of these cells in neurodegenerative disease (ND) studies are their incapability to generate a heterogeneous population of neurons as well as their instability. In addition, most studies reported the use of non-differentiated cells in the Aβ model that do not mimic the *in vivo* scenario. Therefore, it is important to establish a proper cellular model to study the effect of Aβ peptides, which would mimic the normal phenomenon occurring in the *in vivo* environment. Several studies have shown the beneficial effect of stem cells in degenerative diseases due to their capacity to differentiate into any type of cells and their ability to secrete trophic factors that can reverse the damaged tissues. In addition, to the best of our knowledge, there are currently limited data on the neurotoxicity of Aβ peptides in neural cells differentiated from stem cells. In the present study, neural differentiation was carried out using the embryonic stem (ES) cell line, 46C, engineered to monitor the formation of neural precursor cells (NPCs). 46C cell line carries green fluorescent protein (GFP) knocked-in into the SRY-box transcription factor 1 (*Sox1*) open reading frame (ORF) [22]*. Sox1* is a NPC marker and is prominently expressed in proliferating progenitor cells in the mouse embryo during the development of the central nervous system [22-24]. The expression of *Sox1* is silent in undifferentiated ES cells, but is activated upon neural induction and then downregulated during neuronal and glial differentiation [25]. 46C cells are used to facilitate the identification of NPCs, thus allowing us to monitor the success of neural induction protocol at the early stage. Besides, this property enables the purification of both neural and non-neural cells that are generated during neural differentiation of 46C cells for further downstream analysis.

Another important reason to study the cellular model of Aβ is the correlation between neurotoxicity and structural properties of Aβ peptides, which is not completely understood. Previous studies suggested that the size and physicochemical properties of Aβ peptides contribute to the formation and neurotoxicity of insoluble Aβ fibrils. The most common Aβ fibrils found in the senile plaques is Aβ species ending at amino acid 42 (Aβ_42_), which is longer than the rest of Aβ species and more hydrophobic; hence, it is more susceptible to aggregation and toxicity. Meanwhile, Aβ_40_, which is more abundantly produced by the cells than Aβ_42_, is commonly colocalized with Aβ_42_ in the plaque [3,26,27]. Likewise, Aβ_25-35_ fragment can also induce aggregation and toxicity, similar to Aβ_1-42_ [28]. The contributing factors to these phenomena are not known; however, a recent study demonstrated that Aβ_25-40_ fragment, which localizes in the hydrophobic region of the lipid bilayer, could disrupt the phospholipid arrangement in the cell membrane of neurons, leading to the dysregulation of Ca^2+^ ion uptake and subsequent neuronal cell damage [29,30]. Additionally, the abundance and solubility of Aβ peptides are crucial factors of amyloidosis in AD. The soluble aggregated form of Aβ peptides can be easily introduced into the neuronal membrane and can cause neuronal damage and loss [31-34], while the insoluble aggregated form of Aβ peptides, which is deposited in Aβ plaque, exhibits low toxicity [35]. Up until now, there is an ongoing debate about which type and dose of Aβ peptides are the most harmful to neurons. To answer this question, a detailed quantification of Aβ-induced neural cell death and the determination of the onset of toxicity should be carried out.

Here, we aim to assess the neurogenic potential of 46C cells in providing quality neural cells that can be utilized to establish Aβ-peptide-induced oxidative stress model *in vitro*. In this study, the susceptibility of 46C-derived neural cells to Aβ-induced toxicity and oxidative stress was evaluated. Two types of exogenous Aβ peptides, Aβ_1-42_ and Aβ_25-35_, which differ in the length and physicochemical properties, were used. Our results support the hypothesis that Aβ peptides can induce oxidative stress and subsequent neurotoxicity. We observed both Aβ-peptide species to generate significant levels of intracellular ROS, albeit, interestingly, with no effect on promoting cell injury and death in the 46C-derived neural cells. In our opinion, 46 cell-derived neural cells could be useful to establish *in vitro* Aβ-peptide-induced oxidative stress model, suitable for drug screening and fundamental studies, particularly to understand the molecular mechanisms responsible for AD pathogenesis induced by Aβ peptides.

## MATERIALS AND METHODS

### Cell culture

The 46C mouse ES (mES) cells were a gift from Dr. John Mason (University of Edinburgh, UK). The cell line was maintained in Glasgow’s Minimum Essential Medium (GMEM) (BHK-21; Gibco™, USA) supplemented with 10% (v/v) fetal bovine serum (FBS; Gibco, USA); 1% MEM non-essential amino acids (Gibco™, USA), 1 mM sodium pyruvate (Gibco, USA), 0.1 mM 2-mercaptoethanol (Gibco, USA), 2 mM L-glutamine (Gibco ™, USA), and 10 μg/mL human recombinant leukemia inhibitory factor (LIF 1010; Millipore, USA). The cells were seeded at a cell density of 4.0 × 10^4^ cells/cm^2^ into 25 cm^2^ cell culture flasks coated with 0.1% (w/v) gelatin (Sigma, USA). The cells were subcultured every other day when they were 70–80% confluent.

### Neural differentiation assay

Neural differentiation assay was carried out through spontaneous formation of multicellular aggregates, known as embryoid bodies (EBs) using 4-/4+ protocols adapted from a previous study [36]. Briefly, for EBs formation, 5.0 × 10^6^ undifferentiated 46C mES cells were seeded in 100 mm uncoated Petri dish (bacteriological grade) for 4 days in 10 mL media in the absence of LIF and all-trans retinoic acid (ATRA; Sigma, USA) followed by another 4 days in the presence of ATRA. The medium was changed every 2 days. The expression of GFP was assessed every 2 days under inverted fluorescence microscope (OLYMPUS IX51, Germany) to identify *Sox1*-positive cells, signifying NPCs. At the end of the induction period (8^th^ day), the EBs were dissociated with a high concentration of trypsin (4× trypsin-ethylenediaminetetraacetic acid [EDTA] and 4% chicken serum in ×1 phosphate-buffered saline [PBS]) for 5 min in 37°C water bath and agitated to obtain single cells suspension. Single cells were counted prior to plating on the dishes pre-coated with 10 μg/mL of Poly-D-Lysine (PDL; Sigma, USA) in ×1 PBS and 2 μg/mL of laminin from Engelbreth-Holm-Swarm murine sarcoma (Sigma, USA). The cells were seeded at the density of 2.0–3.0 × 10^4^ cells/cm^2^ in N2B27 medium. N2B27 medium was a mixture of Dulbecco’s Modified Eagle Medium [DMEM] (Gibco-Invitrogen, USA) with the ratio 1:1 of DMEM/F12 medium supplemented with N2 (Gibco-Invitrogen, USA). The cells were subsequently incubated at 37°C in 5% CO_2_ incubator. The medium was replaced with fresh N2B27 medium every 2 days. The neural differentiation assay was carried out after 8–9 days post-plating.

### Immunocytochemistry (ICC)

ICC was prepared in 24-well plates. The attached neurons were fixed in 4% paraformaldehyde (PFA; 50 mM NaOH, ×1 PBS) for 30 min, followed by permeabilization in 1% Triton-X 100 in ×1 PBS for 15 min at room temperature (RT). Cells were then incubated in blocking solution (0.3% bovine serum albumin [BSA], 0.1% Tween-20 in ×1 PBS) for 30 min prior to incubation with primary antibody at 4°C overnight. After washing with ×1 PBS, the cells were then incubated with fluorochrome-conjugated secondary antibody immunoglobulin G [IgG] (H+L) for 2 h at RT in the dark. After washing, the cells were counterstained with DAPI (Sigma, USA) for 10 min at RT. The cells were then left in ×1 PBS in the dark until visualization with an inverted fluorescence microscope. The antibodies used in this study are listed in Table 1.

**TABLE 1:**
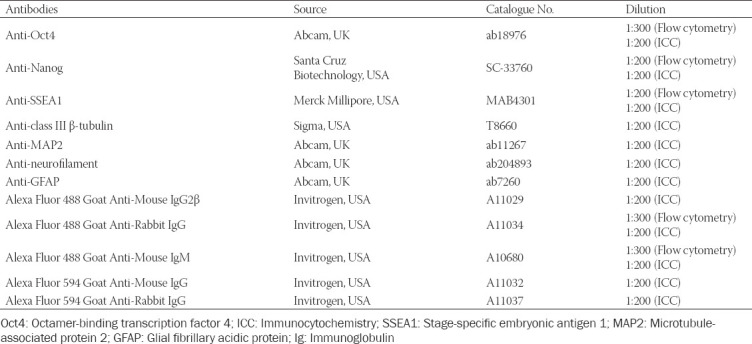
Primary and secondary antibodies and the dilution factors used

### Flow cytometry

#### Pluripotency protein markers

The expression of octamer-binding transcription factor 4 [Oct4] (1:300; Abcam, UK; cat no#ab18976), Nanog (1:200; Santa Cruz Biotechnology, USA; cat no#sc-33760), and stage-specific embryonic antigen 1 [SSEA1] (1:200; Merck Millipore, USA; cat no#MAB4301) was characterized using FACSCanto™ Flow cytometry (BD, USA), to detect pluripotency properties and the stemness of undifferentiated stem cells. The antibodies used in this study are listed in Table 1.

#### NPC marker (Sox1^eGFP^)

Viable *Sox1*^eGFP^-positive cells were sorted by fluorescent activated cell sorting (FACS) in a FACSCanto™ Flow cytometry (BD). Briefly, EBs on day 4, 6, 8, and 10 were dissociated with trypsin (×4 trypsin-EDTA, 4% chicken serum in ×1 PBS) for 5 min in 37°C water bath. Following the dissociation of the cells, the trypsin was deactivated using FACS buffer (10% FBS, in ×1 PBS), and the cells were centrifuged at 800 rpm for 5 min. The cells were washed twice with ×1 PBS and centrifuged again at 800 rpm for 5 min to obtain a cell pellet. Single cells were suspended in FACS buffer prior to FACS analysis.

#### Neuronal protein markers

Immunostaining was performed to characterize neuronal and glial cells in neural cells derived from 46C cells. The neural markers used were: class III, β-tubulin for the detection of post-mitotic neurons (1:200; Abcam, UK; cat no#ab18207); microtubule-associated protein 2 [MAP2] (1:200; Abcam, UK; cat no#ab11267) and neurofilament [NF] (1:200; Abcam, UK; cat no# ab204893) for the detection of mature neurons; and glial fibrillary acidic protein (GFAP) for the detection of glial cells, mainly astrocytes (1:200; Abcam, UK; cat no#ab7260) in 46C-derived neural cells. The antibodies used in this study are listed in Table 1.

### Establishment of in vitro oxidative stress model in neural-derived 46C cell line by Aβ peptides (Aβ_1-42_ and Aβ_25-35_)

#### Preparation of Aβ peptides (Aβ_1-42_ and Aβ_25-35_)

The lyophilized Aβ_1-42_ and Aβ_25-35_ were reconstituted according to the manufacturer’s protocol (AnaSpec, USA) where the peptides were dissolved in 70–100 μL of 1% ammonium hydroxide (NH_4_OH) and ×1 PBS (Gibco, USA) to make 1 mL of 0.2 mM Aβ_1-42_ and Aβ_25-35_ solution. The reconstituted peptides were aliquoted and stored at -80°C for further use.

#### Aβ fibril protein formation

The reconstituted Aβ_1-42_ and Aβ_25-35_ stock was diluted to 100 μM in ×1 PBS. The peptide solutions were then incubated for 24 h with vigorous shaking at 37°C in Orbital Benchtop Shaker (CERTOMAT MO II, Sartorius, France). The fibrils were characterized using transmission electron microscope (TEM) prior to be used for the subsequent experiments.

#### Neurotoxicity of Aβ_1-42_ and Aβ_25-35_ on matured neurons

46C-derived NPCs were seeded in 24-well plates at a cell density of 2.0–3.0 × 10^4^ cells/cm^2^ in N2B27 medium (ratio 1:1). On day 8 of neural induction, the cells were exposed to the Aβ fibril proteins for 24 h. Neurotoxicity was evaluated by 3-(4,5-dimethylthiazol-2-yl)-2,5-diphenyltetrazolium bromide (MTT) colorimetric assay. At post-treatment with Aβ fibril proteins, MTT solution was added into all wells and incubated for 4 h. Following incubation, the mixture of MTT solution and media in the wells were discarded and substituted with 100 mL of dimethyl sulfoxide (DMSO) to solubilize the purple formazan crystal. The cells were then subjected to ELISA microtiter plate reader (AsysHighTech UVM340, Biochrom, UK) at 570 nm wavelength.

#### Analysis of intercellular ROS

Intracellular ROS was measured using OxiSelect Intracellular ROS Assay Kit (Cell Biolabs, USA) according to the manufacturer’s instruction with some modification. The cell permeable 2’,7’-dichlorodihydrofluorescein diacetate (DCFH-DA) diffuses and is deacetylated by cellular esterases to non-fluorescent DCFH which reacts with ROS (includes hydroxyl radical, peroxyl radical, and other ROS activity within a cell) to form a highly fluorescent DCFH. The treated 46C-derived neural cells were washed twice with ×1 PBS followed by preincubation with 200 μL of ×1 DCFH-DA/N2B27 medium for 45–60 min at 37°C. The supernatant was removed, and the cells were washed twice with ×1 Dulbecco’s phosphate-buffered saline (DPBS). The supernatant was then discarded, and the cells were washed twice with ×1 DPBS, followed by the addition of 200 μL of ×2 cell lysis buffer; the mixtures were incubated for 5 min. The mixture (150 μL) was then transferred to a black 96-well plate, and the fluorescence was quantitated using a fluorometric plate reader (FLUOstar Omega, BMG LABTECH, Germany) at 480 nm/530 nm excitation/emission wavelengths.

### Statistical analysis

For statistical analysis, GraphPad Prism version 6.00 for Windows (GraphPad Software, La Jolla California USA) was used. One-way ANOVA and *post hoc* Dunnett’s test (n= 3) were used for cytotoxicity analysis, meanwhile one-way ANOVA and *post hoc* Bonferroni test were used for ROS assay analysis (n =12).

## RESULTS

### Propagation and characterization of 46C cells

**FIGURE 1:**
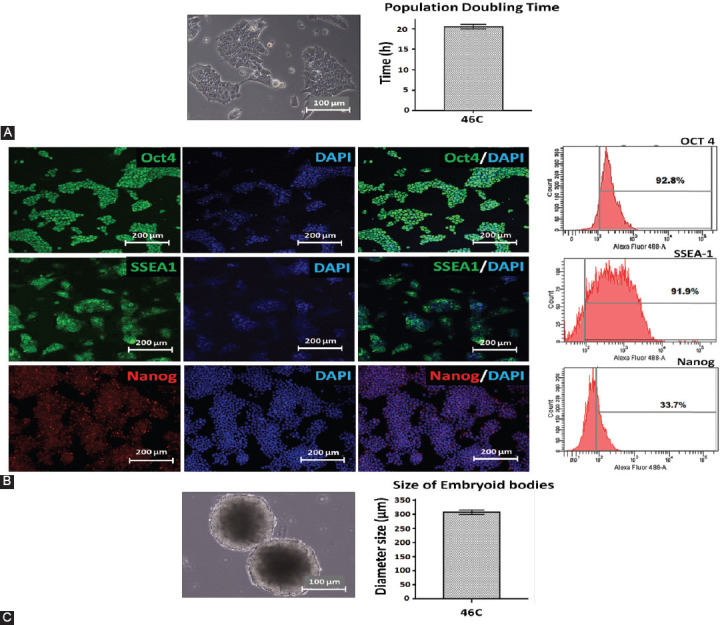
(A) Morphological characteristics and population doubling time of undifferentiated 46C cells. Good quality 46C cells exhibit a high nucleus-to-cytoplasm ratio. The doubling time for 46C cells was 20.0 ± 2.07 h and is representative of randomly chosen passages (n = 12). (B) Immunostaining and flow cytometry analysis of pluripotency-associated protein markers: Oct4, SSEA1, and Nanog. Blue shows nuclear counterstain with DAPI, while green shows expression of Oct4 and SSEA1; red shows expression of Nanog. Flow cytometry analysis of 46C cells for Oct4, SSEA1, and Nanog. The percentage of fluorescent intensity of gated treated cells is shown on the histograms. (C) 46C cell line was able to generate good quality EBs that presented with cavitation process (center), smoothness of the boundary, and acceptable diameter (308.18 ± 28.16 mm; and is representative of randomly chosen passages; n = 12). The scale bars represent 100 mm (A) and (C); and 200 mm (B) for micrographs. Oct4: Octamer-binding transcription factor 4; SSEA1: Stage-specific embryonic antigen 1.

In this study, 46C cells were differentiated into neuronal and glial cells by the 4-/4+ protocol through the formation of multicellular aggregates, i.e., EBs. High-quality 46C cell line is needed for success in neural differentiation assay. The crucial characteristics of 46C cells are their morphology, expression of pluripotency protein markers, and the capability of the cells to form EBs. The morphology of high-quality 46C cells appears to have a high nucleus-to-cytoplasm ratio and a large nucleus with multiple nucleoli (Figure 1A).

46C cells were qualitatively analyzed by ICC and were then quantified by flow cytometry for the expression of three fundamental pluripotency-associated markers, i.e., Oct4, homeobox protein Nanog, and SSEA1. Oct4 and Nanog are transcription factor proteins, which are localized in the nucleus of all types of stem cells. Meanwhile, SSEA1 is an antigenic epitope, also called CD15, which is localized on the cell membrane surface. High expression of these transcription factor proteins and SSEA1 was observed in undifferentiated 46C cells, as shown in Figure 1B, indicating the biological activity and the “stemness” of the cells. DAPI was used to counterstain the nucleus. The quantitative analysis of 46C cells was performed and presented in a histogram. Remarkably, the expression of these pluripotency markers was distinctly high in undifferentiated 46C cells. The 46C cells were observed to express high Oct4 (>90%), Nanog (>30%) and SSEA1 (>90%), indicating high pluripotency of cells. These results may suggest high-potential pluripotent stem cells in cultures.

46C cells underwent spontaneous aggregation to form EBs after 1 day in LIF-free medium. Good-quality EBs presented with the cavitation process, smoothness of the boundary, and acceptable diameter [308.18 ± 28.16 μm; n = 12] (Figure 1C), indicating pluripotency characteristics were observed.

### Neurogenic potential of 46C cells

**FIGURE 2:**
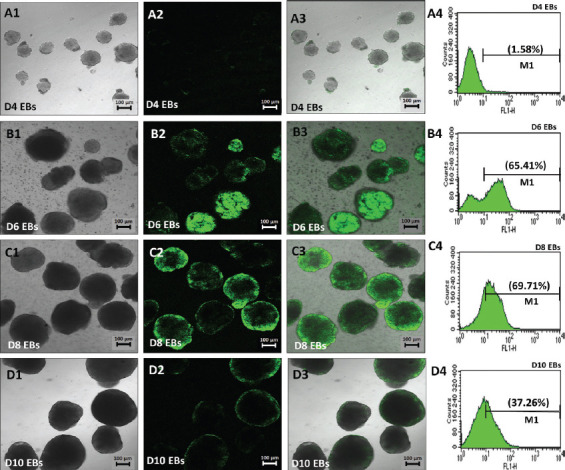
Expression of *Sox1*^eGFP^ during neural differentiation of 46C cells *in vitro* on D4, D6, D8, and D10 EBs. A1, B1, C1, and D1 show phase-contrast pictures of EBs and A2, B2, C2, and D2 fluorescence images of EBs with a clear demonstration of the highest *Sox1*^eGFP^ expression on D8. A3, B3, C3, and D3 demonstrate the merge. A4, B4, C4, and D4 demonstrate the flow cytometry analysis for *Sox1*^eGFP^ expression during neural differentiation of 46C cells showing that D8 EBs have the highest eGFP expression, which is an indicator of neural differentiation success. The scale bars represent 100 mm for micrographs. D: Day; EBs: Embryoid bodies; GFP: Green fluorescent protein.

After 4 days of EB culture, neural induction was performed by the addition of 10 μM ATRA for another 4 days. The qualitative analysis by visualization of *Sox1*^eGFP^ expression under inverted fluorescence microscope showed the highest intensity of GFP on D8 EBs, supported by the quantitative analysis using flow cytometry (Figure 2B).

**FIGURE 3:**
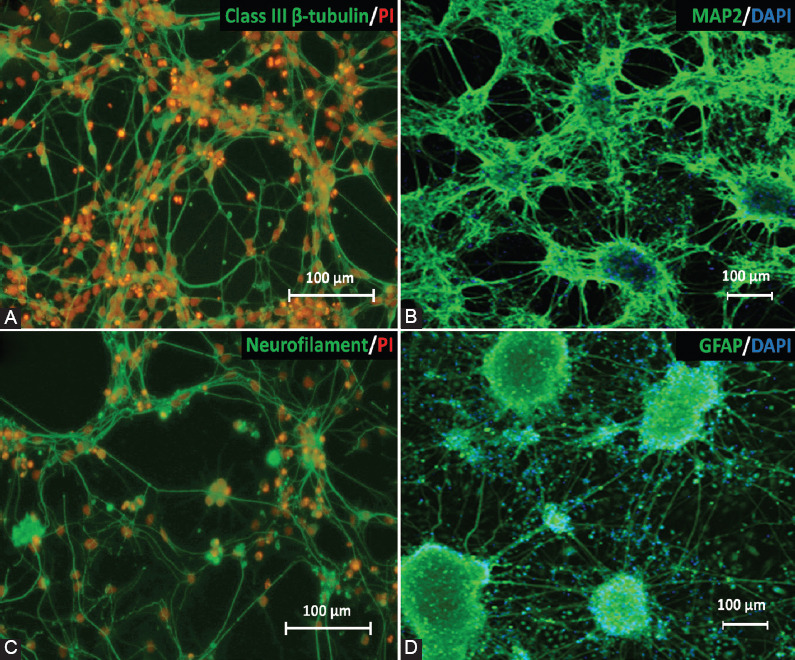
Immunocytochemical analysis was performed to evaluate the expression of neural and glial cell proteins after neural differentiation of 46C cells on day 5 neuron post-plating on PDL/laminin-coated plate. The expression of (A) post-mitotic neurons (class III β-tubulin); (B) mature neurons (MAP2); (C) neurofilament; and (D) astrocytes (GFAP) was observed in the neuron cultures. Green indicates the expression of protein markers; meanwhile, nuclei were counterstained with PI (red) or DAPI (blue). Scale bar is 100 mm. PDL: Poly-D-Lysine; MAP2: Microtubule-associated protein 2; GFAP: Glial fibrillary acidic protein; PI: Propidium iodide.

On D8 EBs, *Sox1*^GFP+^ EBs were dissociated to single cells and re-plated on PDL/lysine-coated plates in the presence of N2B27 medium. Our findings demonstrated that a number of mature neuronal subtypes and glial cells were efficiently generated from 46C cells (Figure 3), thus providing an ideal *in vitro* model. *Sox1*^eGFP+^ cells started to form neural-like structures on day 2 post-plating and continued to differentiate until day 6 post-plating, with more prominent mature neurons observed (Figure 3A-C). We also found that 46C cells were capable to differentiate into astrocytes, as marked by the expression of GFAP (Figure 3D).

### Establishment of in vitro oxidative stress model in neural-derived 46C cells by Aβ peptides (Aβ_1-42_ and Aβ_25-35_)

#### Ab (Aβ_1-42_ and Aβ_25-35_) fibril protein formation

**FIGURE 4:**
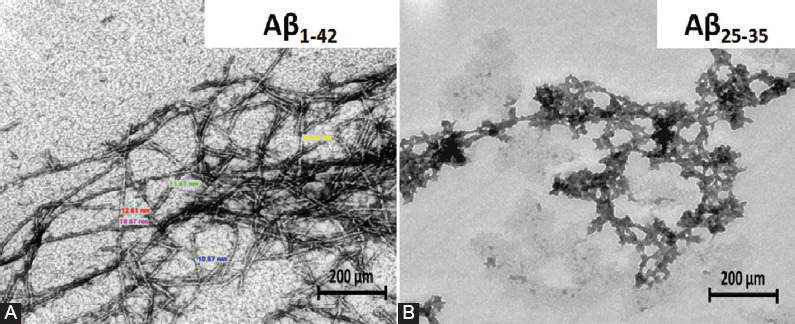
Morphological appearance of amyloid beta (Aβ) fibrils under transmission electron microscope. Aβ fibril aggregates formed from Aβ_1-42_ peptide demonstrating a “striated ribbon” morphology, with a diameter range from 6 to 15 nm (A), while, Aβ_25-35_ peptide formed tiny and short Aβ aggregates (B). Scale bar is 200 nm.

Our Aβ_1-42_ preparation showed a highly homogenous morphology of fibril aggregates as observed under TEM. The fibrils exhibited a “striated ribbon” morphology, characterized by long, thin and straight unbranched fibrils, with a diameter of 6–15 nm, which resembled mature fibrils (Figure 4A). Meanwhile, the dissolution of Aβ_25-35_ resulted in rapid aggregation, giving rise to tiny and short Aβ aggregates, which resembled protofibril-like assemblies (Figure 4B).

#### Assessment of cell viability by MTT assay and intracellular ROS by DCFH-DA assay

**FIGURE 5:**
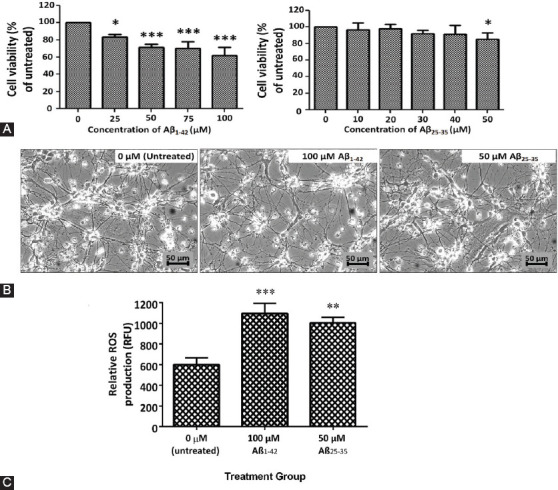
(A) Neural-like cells derived from 46C cells were exposed to different concentrations of Aβ_1-42_ and Aβ_25-35_ fibrils for 24 h. About 25–100 μM of Aβ_1-42_ fibrils induced a significant decrease in cell survival in a dose-dependent manner as compared to the control, while 50 μM of Aβ_25-35_ fibrils decreased the cell viability significantly to 85%, though the IC_50_ was not obtained. All data are expressed as mean ± SD (n = 3), where *p < 0.05 and ***p < 0.001 vs. untreated group (one-way ANOVA and *post hoc* Dunnett’s test). (B) Neural cells were not easily damaged by Aβ peptides, and neural cells remained intact when compared to untreated neural cells. Scale bar is 50 μm. (C) About 100 μM of Aβ_1-42_ and 50 μM of Aβ_25-35_ induced a significant increase in reactive oxygen species (ROS) levels as compared to untreated control. All data are expressed as mean ± SEM (n = 12), where **p < 0.01 and ***p < 0.001 vs. untreated group (one-way ANOVA and *post hoc* Bonferroni test).

To confirm that Aβ peptides mediated the toxicity, we evaluated the viability of 46C-derived neurons by MTT assay. The cytotoxicity of Aβ_1-42_ peptide started at a concentration of 25 μM, resulting in minimal toxicity to 46C neural cells with approximately 80% survival (*p* < 0.01), while 50 μM Aβ_25-35_ peptide exhibited approximately 15% of cytotoxicity (*p* < 0.05), signifying minimal injury. Interestingly, treatment with a high concentration of Aβ_1-42_ (100 μM) still failed to cause 50% of neural cell death; approximately up to only 40% (*p* < 0.001; Figure 5A). There was no observable cellular death or damage and were only insignificant morphological alterations, as seen by microscopy analysis in 46C-derived neural cells treated with 100 μM Aβ_1-42_ and 50 μM Aβ_25-35_ (Figure 5B). The production of intracellular ROS was monitored by measuring the fluorescence intensity from the reaction of intracellular ROS with DCFH-DA using fluorescence microplate assay. Surprisingly, the treatment with 100 μM Aβ_1-42_ resulted in a significant, 1.8-fold, increase (*p* < 0.01) in intracellular ROS activity, whereas the treatment with 50 μM Aβ_25-35_ resulted in 1.7-fold increase (*p* < 0.01) in intracellular ROS activity as compared to untreated neurons (Figure 5C).

## DISCUSSION

We have developed a novel Aβ-induced oxidative stress AD model in 46C-derived neural cells. The neurotoxicity of Aβ peptides in the form of extracellular fibrillar aggregates *in vitro* has been well-documented in the previous studies using either primary neuronal cells or cancerous cell lines. We proposed that stem cell lines may provide a more reliable source of neurons and glial cells as they represent a normal condition prior to the development of the pathogenicity of AD. In the present study, transgenic mouse ES (46C) cell line was used for the establishment of Aβ-induced oxidative stress AD model.

ES cells are pluripotent cells derived from either the dissociated morula [37], intact blastocyst [38], or the inner cell mass (ICM) of the early-stage embryo [39]. However, in most reported studies, mouse ES cells were isolated from E3.5 ICM of the preimplantation embryo [40,41]; meanwhile, human ES cells were isolated from E6 ICM of the blastocyst from *in vitro* fertilization [42]. Mouse ES cells were cultured in the presence of LIF [41,43] or mouse embryonic fibroblast [42,44], which promote self-renewal while maintaining their undifferentiated state. ES cells have two distinct properties. First, ES cells can be grown under defined conditions to maintain their undifferentiated and pluripotent nature, thus, they can self-renew indefinitely. Second, they can be stimulated with biochemical and physical stimuli to differentiate into a variety of cell lineages [45], including neurons and glial cells [46]. Several studies have shown the beneficial effect of stem cells in degenerative diseases due to their capacity to differentiate into any type of cells and their ability to secrete trophic factors that can reverse the damaged tissues. Therefore, there is a considerable interest in ES cells as a potential source of transplantable cells for cellular and genetic therapies of ND such as AD and Parkinson’s disease, as well as for the development of *in vitro* models for drug testing and toxicological screening.

46C cells are a transgenic mouse ES cell line, which was transduced with the *GFP* gene, introduced into the *Sox1* ORF. In the present study, *Sox1*, the marker for NPCs, was used to monitor the effectiveness of neural differentiation of 46C cells. The GFP expression marks the activation of *Sox1* and hence the presence of NPCs. The initial characterization of 46C cells was based on their morphology and pluripotency status to ensure the identity and purity of our stem cells in culture prior to the development of neural cell profiling and disease modeling. Oct4, Nanog, and SSEA1 are three pluripotency markers that have been found to be involved in self-renewal and in maintaining the pluripotency of ES cell phenotype [47,48]. These three markers were chosen to assess the stemness and pluripotency of 46C cells in this study. As shown by ICC (Figure 1B), both Oct4 and Nanog expression were obviously expressed and localized in the nucleus of 46C cells, indicating the biological activity that is regulated in the cell nucleus, while SSEA1 expression was localized on the cell surface. Quantitatively, the flow cytometry analysis showed that 46C cells exhibited high expression levels of Oct4, Nanog, and SSEA1. The transcription factor Oct4, encoded by the gene *POU5F1*, is expressed by totipotent and pluripotent cells especially during early embryogenesis, mainly by the cells of the morula and ICM of early blastocyst stages. In previous studies, after implantation, Oct4 expression persisted in the epiblast, and consequently was downregulated during gastrulation. In the later stages, Oct4 expression could only be observed in primordial germ cells [49,50]. *In vitro*, Oct4 is also present in undifferentiated ES and embryonal carcinoma (EC) cells, marking the pluripotency of ES and EC cells in culture. Oct4 expression is downregulated in ES and EC cells upon the removal of LIF. Two isoforms are characterized for Oct4 protein: 1) Oct4A, localized in the nuclei of cells and highly expressed in ICM and ES cells. Oct4A marks the pluripotency of cells; 2) Oct4B, localized in the cytoplasm of cells and also in the nuclei of blastomeres prior to compaction into the morula. However, the function of Oct4B is still unknown [51].

Nanog is a transcription factor that belongs to the homeobox DNA binding family and is essential for the formation of germ cells. Similar to Oct4, Nanog expression is found during early embryogenesis and is localized in the nuclei of cells. Nanog protein is responsible for ICM to differentiate into epiblast and maintain its pluripotency [52,53]. The expression of Nanog is capable to promote self-renewal and pluripotency in ES cells independently of LIF [52]; however, the expression is downregulated rapidly as ES cells differentiate [54]. SSEA1 proteins are only expressed in murine EC cells, murine ES cells, and murine and human germ cells, but not in human ES cells and induced pluripotent stem cells [55,56]. SSEA1 proteins are identified during preimplantation of the eight-cell stage mouse embryo [57], and the expression drops in the postimplantation embryo, where it is only found localized in the brain and primordial germ cells [58]. *In vitro*, SSEA1 expression is upregulated in undifferentiated ES cells, but downregulated in differentiated ES cells, upon the removal of LIF and the addition of retinoic acid (RA) [59,60]. Therefore, we suggest that all the protein markers used in the present study are required to maintain 46C cell pluripotency in culture.

Another important indication of pluripotency is the ability of cells to differentiate into derivatives of the three germ layers: ectoderm, endoderm, and mesoderm. Our results showed that 46C cells were able to form good quality multicellular aggregates known as EBs, using spontaneous differentiation method, the 4-/4+ protocol; overall indicating germ layer differentiation. 46C cells generated good quality EBs that presented with the cavitation process (center), smoothness of the boundary, and acceptable diameter size, ranging from 100 to 300 μM (Figure 1C). Due to similarities between EBs and pre-gastrulation embryos, several EB-based protocols (spontaneous differentiation) have been adopted to differentiate ES cells into different types of cells. The ability of EBs to generate three primary germ layer-derived cells has made it a standard parameter to examine the pluripotency status of stem cells *in vitro*.

The present study confirmed the ability of 46C cells to differentiate into neural lineage through the formation of multicellular aggregates, EBs, using the 4-/4+ protocol [36], in the absence of LIF and with the addition of RA as the neural stimulant [61]. Our findings showed that *Sox1*^GFP+^ cells were first detected in 46C EBs on day 6 following neural induction, which indicated the presence of NPCs in our cultures (Figure 2B). We used FACS to assess the number of *Sox1*^GFP+^, following the 4-/4+ protocol. We found that a major population of *Sox1*^GFP+^ cells was detected approximately on day 8 of 46C EBs (Figure 2C), and a small population of *Sox1*^GFP+^ cells was detected before day 4 (Figure 2A) and after day 10 (Figure 2D) of 46C EBs. Our data are in agreement with the previous work by Nordin et al. [62]. Day 8 EBs were then dissociated into single cells and re-plated onto PDL/laminin-coated dish. Serum-free media supplemented with N2 and B27 (N2B27 medium) were used in this neural induction protocol. Morphologically, this protocol revealed a heterogeneous population of neuronal and glial cells in the culture, which mimicked the *in vivo* environment. The neurogenic potential of 46C cells was confirmed by immunostaining analysis with the class III β-tubulin TUJ1, a marker for post-mitotic neurons (Figure 3A), MAP2 and NF markers for mature neurons (Figure 3B and C, respectively), as well as with GFAP, a marker for glial cells, primarily astrocytes (Figure 3D). A major population of TUJ1-positive cells, MAP2- and NF-positive cells, as well as a small population of GFAP-positive cells, were found in our culture. In our experience, the 4-/4+ protocol was able to efficiently generate neurons and neuron supporting cells from transgenic 46C cells, thus providing an ideal *in vitro* model that mimics an *in vivo* phenomenon, suitable for drug screening and brain studies.

We further explored the neurotoxicity effects of Aβ peptides in 46C-derived neural cells. The deposition of Aβ peptides into fibrillar aggregates plays a critical role in the onset of pathological events in AD. Different Aβ preparations, namely Aβ_1-42_ and Aβ_25-35_ peptides, were assessed for their ability to induce toxicity in 46C-derived neural cells. In the present study, the structure of Aβ fibril morphologies formed under physiologically appropriate pH conditions was compared between Aβ_1-42_ and Aβ_25-35_ peptides. Using TEM, we found that the fibrils of Aβ aggregates formed from Aβ_1-42_ peptides demonstrated normal, elongated fibrillar morphology with a diameter of 6–15 nm, which resembled mature fibrils (Figure 4A). On the contrary, Aβ_25-35_ peptides formed tiny and short Aβ aggregates, which resembled protofibril-like assemblies (Figure 4B). To confirm Aβ-mediated neurotoxicity, cell viability by MTT assay was carried out in 46C-derived neural cells. To access the effect of concentration on cell viability, 46C-derived neural cells were treated for 24 h with increasing doses of Aβ_1-42_ and Aβ_25-35_ fibrils, ranging from 25 to 100 μM and 10 to 50 mM, respectively (Figure 5A). The results showed that the Aβ fibrils influenced cell viability in a dose-dependent manner. It was expected that the cell viability would drop significantly when exposed to high concentrations of Aβ_1-42_ and Aβ_25-35_ fibrils. Although there was a decline in cell viability IC_50_, the treatment concentration that kills 50% of neural cells, was not observed in neither of the treatments. Using the highest tested concentrations (50 μM of Aβ_25-35_ and 100 μM of Aβ_1-42_ fibrils), we did not observe any obvious toxicity effects of the exogenous Aβ fibrils on the morphology of the neurons (Figure 5B). However, it should be noted that 40% of neuronal mortality *in vivo* could be catastrophic for the brain. Another important finding was that the toxicity of Aβ fibrils dramatically increased ROS generation in 46C-derived neural cells post-treated with these Aβ peptides for 24 h (Figure 5C). Our results indicate success in establishing *in vitro* oxidative AD model using 46C cells, by inducing the generation of ROS.

The most well-known theory of Aβ-induced neurotoxicity hypothesizes that neuronal cell death in AD brain results from the accumulation of Aβ fibrils, which is associated with oxidative stress. Aβ fibrils have the potential to initiate and generate ROS in the brain [8,11,63-65]. Nevertheless, there are debatable issues that have been questioned in several studies regarding oxidative stress in AD, i.e., whether it is the cause or the consequence of the accumulation of Aβ fibrils in AD brain. In this regard, Pappolla et al. investigated the onset of oxidative injury in the brain, whether it happens before or after Aβ fibrils accumulation. They provided evidence showing that the oxidative stress markers superoxide dismutase and heme oxygenase-1 (HO-1) are found following Aβ peptide deposition in the brain cortical area of aged transgenic (Tg^+^) AD mice model as compared to normal mice (Tg^−^). Moreover, the oxidative stress markers were observed in aged Tg^+^ mice even though the senile plaques were not developed yet. They suggested that Ab oligomeric and “pre-mature” senile plaques might also stimulate oxidative stress response at the minimum level. The oxidative stress markers were absent in young Tg^+^ mice [66]. In the present study, we showed that both Aβ_25-35_ and Aβ_1-42_ peptides could induce intracellular ROS production in 46C-derived neural cells. The 42 amino acid fragment of Aβ_1-42_ peptide has been shown to exhibit neurotoxicity *in vitro* and *in vivo*. Aβ_42_ fragments are commonly deposited in the plaque and are found in AD brain. The accumulation of Aβ_1-42_ peptides in neurites resulted in the formation of neuritic plaque and ultimately neurite degeneration [67]. Furthermore, several evidences show that Aβ peptides induce oxidative stress. Previous studies reported that Aβ_1-42_ peptide significantly increased ROS production in several cell-line models, including PC12, SH-SY5Y and SK-N-SH cells [68]. In particular, Aβ_1-42_ peptide has been shown to induce lipid peroxidation, as reported by Cetin et al., who administered Aβ_1-42_ peptide via intracerebroventricular injection in young and aged rats. They found a significant increase in malondialdehyde, a marker for lipid peroxidation, in the young and aged rats [69]. Although the 11 amino acid fragment of Aβ_25-35_ peptide has a short-length monomer, it has often been studied as a powerful neurotoxicity inducer, similar to Aβ_1-42_ peptide. Zhang et al. reported a significant increase in ROS level in human neuroblastoma cells (SH-SY5Y) treated with Aβ_25-35_ peptide. They also found that the treatment with Aβ_25-35_ peptide resulted in the alteration of the levels of the antioxidant enzymes thioredoxin, HO-1, and peroxiredoxin, suggesting that Aβ peptides induce oxidative stress in neuronal culture [14]. Similar findings have been reported in rat PC12 cells [18-20] and rat hippocampal cells [70]. Together, our findings and those described above suggest that the increase in intracellular ROS levels in Aβ-induced 46C-derived neural cells might be a possible mechanism involved in the neurotoxicity.

Despite the similarities observed in the properties of Aβ_25-35_ and Aβ_1-42_ peptides, there are several differences between these two peptides. First, these peptides are derived from different regions of APP; Aβ_1-42_ peptide is derived from the N-terminal region of the transmembrane domain, while Aβ_25-35_ peptide is derived from the central region and localizes in the hydrophobic core of the membrane bilayer. Second, due to their strategic localization in the cell membrane, both peptides exert specific toxicity effects. The abundance of N terminal-Aβ_1-42_ fragment is crucial for Aβ peptide aggregation and association to the membrane, while the central-Aβ_25-35_ fragment is important for intracellular Ca^2+^ ion regulation and may lead to synaptic toxicity and failure [28]. Finally, it has been reported that the mechanism of oxidative stress induced by Aβ_25-35_ peptide may be different from that of Aβ_1-42_ [71].

Although we were able to produce Aβ-induced oxidative stress in 46C-derived neural cells, we also found inconsistencies between our and the previous studies. Our findings demonstrate a significant increase in intracellular ROS levels upon treatment with both types of Aβ peptides, even though the accumulation of exogenous Aβ peptides seems to have no effect on promoting cell injury and death in our 46C-derived neural cell model. This may be due to several reasons. The exogenous Aβ peptides may act in different ways as compared to the endogenous Aβ peptides. Besides, given the fact that high concentrations of Aβ peptides were needed to induce neurotoxicity in the present study, we hypothesize that the presence of glial cells (mainly astrocytes) in our culture may have strengthen the neurons and protected them from the toxicity of Aβ peptides, which resembles the common *in vivo* scenario.

## CONCLUSION

Our results provide preliminary evidence of Aβ-induced toxicity in cultured neural cells differentiated from stem cells through the generation of intracellular ROS. We showed that 46C-derived mature neurons are stable neurons and that their cell viability and integrity cannot be easily challenged by the toxicity of Aβ-induced oxidative stress. Thus, further investigations on the molecular mechanisms, the optimal dosage, and the duration of exposure responsible for the neurotoxicity induced by Aβ peptides in 46C-derived neural cells are recommended. Nevertheless, we foresee stem cell-derived neural cells as a valuable and promising cell source in providing an ideal *in vitro* oxidative stress model that mimics the *in vivo* pathogenesis of NDs, particularly AD.
